# Optimizing maturity and dose of iPSC-derived dopamine progenitor cell therapy for Parkinson’s disease

**DOI:** 10.1038/s41536-022-00221-y

**Published:** 2022-04-21

**Authors:** Benjamin M. Hiller, David J. Marmion, Cayla A. Thompson, Nathaniel A. Elliott, Howard Federoff, Patrik Brundin, Virginia B. Mattis, Christopher W. McMahon, Jeffrey H. Kordower

**Affiliations:** 1 Department of Neurological Sciences, Rush University Medical Center, Chicago, IL USA; 2 Department of Neurobiology, Barrow Neurological Institute, Phoenix, AZ USA; 3 FUJIFILM Cellular Dynamics Inc., Madison, WI USA; 4 Brooklyn ImmunoTherapeutics, Brooklyn, NY USA; 5 Parkinson’s Disease Center, Department for Neurodegenerative Science, Van Andel Institute, Grand Rapids, MI USA; 6 ASU-Banner Neurodegenerative Disease Research Center and School of Life Sciences, Arizona State University, Tempe, AZ USA

**Keywords:** Induced pluripotent stem cells, Parkinson's disease, Stem-cell therapies

## Abstract

In pursuit of treating Parkinson’s disease with cell replacement therapy, differentiated induced pluripotent stem cells (iPSC) are an ideal source of midbrain dopaminergic (mDA) cells. We previously established a protocol for differentiating iPSC-derived post-mitotic mDA neurons capable of reversing 6-hydroxydopamine-induced hemiparkinsonism in rats. In the present study, we transitioned the iPSC starting material and defined an adapted differentiation protocol for further translation into a clinical cell transplantation therapy. We examined the effects of cellular maturity on survival and efficacy of the transplants by engrafting mDA progenitors (cryopreserved at 17 days of differentiation, D17), immature neurons (D24), and post-mitotic neurons (D37) into immunocompromised hemiparkinsonian rats. We found that D17 progenitors were markedly superior to immature D24 or mature D37 neurons in terms of survival, fiber outgrowth and effects on motor deficits. Intranigral engraftment to the ventral midbrain demonstrated that D17 cells had a greater capacity than D24 cells to innervate over long distance to forebrain structures, including the striatum. When D17 cells were assessed across a wide dose range (7,500-450,000 injected cells per striatum), there was a clear dose response with regards to numbers of surviving neurons, innervation, and functional recovery. Importantly, although these grafts were derived from iPSCs, we did not observe teratoma formation or significant outgrowth of other cells in any animal. These data support the concept that human iPSC-derived D17 mDA progenitors are suitable for clinical development with the aim of transplantation trials in patients with Parkinson’s disease.

## Introduction

As the population continues to age, a pandemic of Parkinson’s disease (PD) is emerging, with conservative estimates of over 14 million victims globally by 2040^1^. While PD patients display a wide range of non-motor features, the defining symptoms are progressive motor deficits due to striatal dopaminergic insufficiency secondary to loss of dopaminergic nigral neurons. Current therapies are symptomatic, mostly focused on ameliorating motor deficits. Since no current therapy arrests or reverses the disease process, there is a major unmet need for new and effective PD treatments. The principal pharmacologic therapies for PD are oral L-DOPA or dopaminergic agonists, which initially provide potent relief from motor symptoms, but after 5–10 years most patients experience debilitating motor fluctuations and dyskinesias^2^. Deep brain stimulation of the subthalamic nucleus (STN) or internal segment of the globus pallidus provides an alternate and effective approach to treat PD motor symptoms, but is primarily indicated for younger patients who do not display cognitive decline and requires periodic battery changes. An alternative approach is the transplantation of midbrain dopaminergic (mDA) cells which could offer a more physiologically relevant delivery of dopamine and confer functional benefits similar to L-DOPA, but without the adverse effects^3,4^.

A large body of work has demonstrated that rodent and human fetal ventral mesencephalic (hfVM) dopamine neurons survive well, innervate the host and form synapses, release dopamine, and alleviate motor deficits when grafted to the dopamine-depleted striatum of experimental animals^5,6^. Some patients in open-label hfVM trials^7,8^ exhibited clinical improvement. However, randomized double blinded, placebo-controlled, clinical trials indicated that these benefits were too variable to meet the trials’ primary endpoints, although predefined secondary endpoints (Unified Parkinson’s Disease Rating Scale, UPDRS) showed statistically significant benefits in younger (<60 years of age; ref. ^9^) or less impaired (UPDRS in off <49; ref. ^10^) subjects. Additionally, some patients developed graft-induced dyskinesias (GID)^9,11,12^, possibly related to pre-existing L-DOPA-induced dyskinesias and the transplants containing serotonergic cells alongside the desired dopaminergic neurons^13,14^. These findings prompted a re-evaluation of the approach. More recently, the European collaborative consortium, TRANSEURO, revisited fetal transplantation in an open-label trial (NCT01898390) with 11 patients at relatively early disease stages who had not developed significant L-DOPA-induced dyskinesias prior to grafting^15^.

The isolation of human embryonic stem cells (hESCs)^16^ and subsequent development of protocols to generate iPSCs^17^ allowed for the manufacturing of various adult cell types for therapeutic applications, unconstrained by the practical and ethical complexities of sourcing aborted fetal tissue. Studies utilizing hESCs^18–20^ or iPSCs^21–24^ have shown that PSCs can be differentiated into mDA neurons and reverse motor deficits in animal models of PD. As a renewable tissue source with the potential for “off-the-shelf” dosing and better batch-to-batch consistency, PSCs are a promising cellular substrate for mDA neurons as an alternative to hfVM tissue.

We have previously shown that iPSC-derived mDA neurons, differentiated via a floor plate intermediate, engraft, survive long-term, and reverse drug-induced motor asymmetry in athymic rats with unilateral 6-hydroxydopamine (6-OHDA) lesions^21,22^. However, innervation of the striatum by these cells was modest compared to published studies using fetal tissue^25–27^. Across many studies, cells in various stages of mDA development have been transplanted, and the most robust engraftment and functional recovery has been observed when using progenitors or immature neurons^18,28–30^.

Therefore, in developing our robust, clinically-compliant, mDA differentiation process, the present paper examined the engraftment, innervation, and functional efficacy in hemiparkinsonian rats using cells from different developmental stages. Specifically, we compared cells considered mDA progenitors (cryopreserved on Day 17 (D17)), immature mDA neurons (D24), and purified mDA neurons (D37), comparing them to R&D grade purified mDA neurons (D38, G418) that have been characterized previously and are available commercially^21,22^. Additionally, we explored whether D17 or D24 cells can provide long-distance innervation by grafting them into the substantia nigra (SN). Finally, to further characterize the performance of D17 mDA progenitors, which we had found to have the most robust survival and fiber outgrowth, we conducted a dose-ranging study and determined the lowest dose that exerted an early onset of functional recovery in hemiparkinsonian rats.

## Results

### Characterization in vitro

**Fig. 1:**
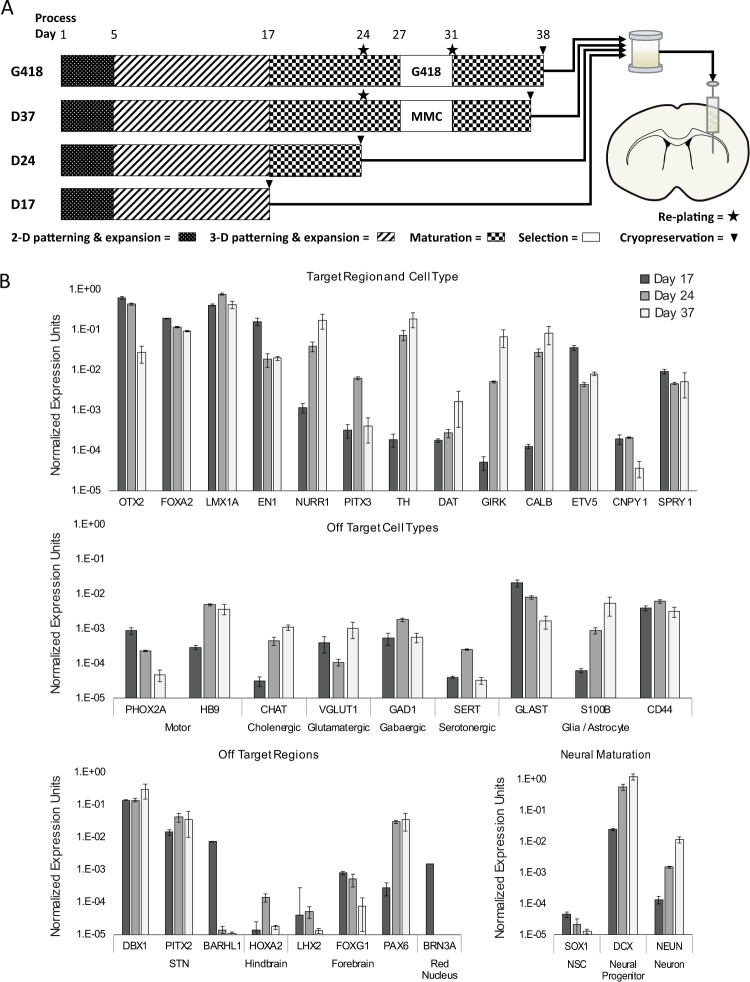
Differentiation and gene expression in vitro. **A** Schematic representation of differentiation and transplantation. MMC = mitomycin c. **B** qPCR comparing mRNA expression at iPSC-mDA differentiation Days 17, 24, and 37 of target and off-target regional, cell type, and neural maturation markers. Three biological replicates were analyzed in technical triplicate for each process time point. Mean Ct values are expressed as relative to glyceraldehyde‐3‐phosphate dehydrogenase (GAPDH) (ΔCt). Error bars are SEM. Significance indicated in Supplementary Table 3.

Our previous transplantation studies utilized research-grade iPSC-derived mDA neurons, and cells made using variations of the same differentiation protocol^21,22^. For the next stages in the development of a cell therapy, we undertook the transition to a process suitable for cGMP manufacturing and clinical trial. The starting material was shifted to a clinical grade human iPSC line. An iPSC master cell bank and working cell bank were manufactured under cGMP conditions. The early stage of iPSC-mDA differentiation was adapted by altering the timing and concentration of small molecule inhibitors. To address safety and regulatory concerns, the raw materials used in the differentiation process were clinical grade where possible. The iPSC-mDA neurons differentiated to the most advanced maturational stage (D37) were enriched during the differentiation process using a low concentration of mitomycin C to remove proliferative cells as previously described^22^ (Fig. 1A). This approach bypassed the need for the drug selection gene cassette used in the R&D grade G418 cells. The mDA progenitor (D17) and immature (D24) mDA neurons cannot be enriched with mitomycin C because they are still proliferative; thus a major goal of this study was to demonstrate that the adapted differentiation process (without an enrichment step) was adequate to prevent unwanted cell proliferation in grafted D17 and D24 cells.

Our previous studies demonstrated that the human iPSC-mDA neurons express high levels of regional midbrain markers and low levels of forebrain and hindbrain markers^21,22^. A similar gene expression panel was used to characterize cells made using the differentiation process adapted for translational use (Fig. 1B and Supplementary Table 1). All differentiation stages (Days 17, 24, and 37) highly expressed regional midbrain markers *OTX2*, *FOXA2*, and *LMX1A*. *EN1* was most highly expressed at D17 then decreased by D24 and maintained that level of expression through D37. More mature mDA markers (*NURR1*, *TH*, *DAP*, *GIRK*, *CALB*) were either expressed at very low levels or not at all on D17 and showed a progressive increase from D24 to D37. *PITX3* expression was highest at D24. Markers reported to be predictive of good engraftment^31^, *ETV5* and *SPRY1*, were expressed at all stages, while *CNPY1* had low expression at D17 and D24 and was nearly undetectable by D37. Expression levels of markers for non-mDA cell types such as motor (*PHOX2A*, *HB9*), cholinergic (*CHAT*), glutamatergic (*VGLUT1*), GABAergic (*GAD1*), and serotonergic (*SERT*) neurons were at the limit of detection or not expressed across all differentiation stages. The most highly expressed off-target marker was *GLAST*, indicating that some astrocyte precursors were present in the culture. Consistent with the presence of STN neurons, which bear semblance to mDA neurons in terms of expression of some molecular markers^32,33^, we observed expression of *DBX1*, *PITX2*, and *BARHL1* at all stages of differentiation. The hindbrain marker *HOXA2* was not expressed, and low levels of forebrain markers were detected throughout D17-37. However, flow cytometry demonstrated that <1% of D17 cells express FOXG1 or PAX6, indicating a lack of forebrain neuron progenitors (data not shown). We also detected *BRN3A* which is expressed in the red nucleus in the midbrain^34,35^. At all time points (D17, D24, and D37) the marker of neural stem cells *SOX1* was not expressed, indicating that the cultured cells had passed the stem cell stage of differentiation. At each of the three time points the neural progenitor marker *DCX* was expressed, while expression of the more mature neural marker *NEUN* increased from D17 to D37.

**Fig. 2:**
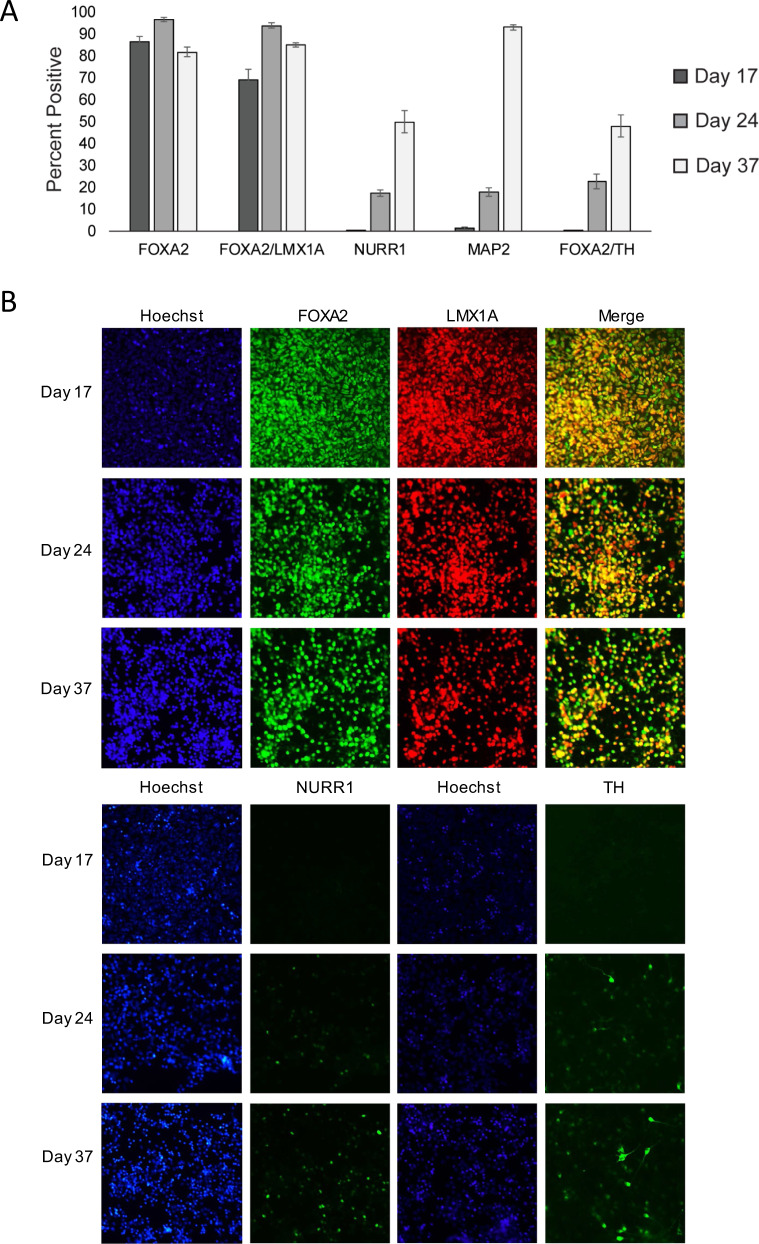
Protein expression in vitro. **A** Flow cytometry comparing immunoreactive populations at iPSC-mDA differentiation Days 17, 24, and 37 of mDA target markers. Quantification of positive cell populations of live cells shown for FOXA2+, FOXA2+/LMX1+, NURR1+, MAP2+, and FOXA2+/TH+. Three biological replicates were analyzed for each time point (Mean ± SEM). **B** Immunocytochemistry comparing immunoreactive populations at iPSC-mDA differentiation Days 17, 24, and 37 of mDA target and off-target markers. Images are representative of three biological replicates analyzed for each time point.

Next, we used flow cytometry to examine the mDA population at the protein level (Fig. 2A) and single cell PCR to examine the mDA population at the RNA level (Supplementary Fig. 1). The percentage of FOXA2-immunoreactive (ir) cells remained high (>80%) from D17 through D37, while co-expression of FOXA2 and LMX1 was around 70% at D17, increasing above 90% by D24. This population of FOXA2/LMX1-ir cells remained high (~85%) in D37 cultures. As expected and consistent with the qPCR results, more mature markers such as NURR1, MAP2, and TH were not detected in D17 samples. The total population percentages of each of these three markers increased over time with approximately 20% being immunoreactive for each in D24 samples, and 50% (NURR1, FOXA2/TH) or 90% (MAP2) being immunoreactive in D37 samples. Immunocytochemistry was used to visually identify these populations of cells (Fig. 2B and Supplementary Fig. 2). Consistent with the flow cytometry results, LMX1A and FOXA2 were co-expressed in a high percentage of cells at each developmental time point. Also consistent with the flow cytometry, NURR1- and TH-ir cells were not present at D17, while a smattering was seen by D24, and a higher number of cells, as well as brighter individual cells, were observed at D37. MAP2 was not detected in D17 samples but became increasingly expressed over time with robust MAP2-ir at D37. Inversely, Nestin-ir cells were abundant on both D17 and D24, but nearly undetectable at D37. STN markers BARHL1 and PITX2 were detected at all time points with few immunoreactive cells present at D17 and an increasing number of cells detected over time. A small percentage of the D37 cells express BARHL1, suggesting that STN neurons are a minority subset of the NURR1-ir cells, and are significantly outnumbered by immature mDA neurons.

Together, these data show that our differentiation protocol produces cultures with a primarily midbrain phenotype that include cells from regions close to the SN, including the STN and red nucleus. Furthermore, there was very little contamination of forebrain or hindbrain cells. Finally, D17 cells are at a progenitor stage and do not express NURR1 or other markers characteristic of mDA neurons, other than EN1.

### Effect of cellular maturity on transplant survival and function

To evaluate the effect of cellular maturity on transplant survival, we injected grafts of D17, D24, D37, or G418 cells into the striatum bilaterally of intact athymic rats. At 3 months post-transplantation (Supplementary Fig. 3), coronal sections stained for human-specific neural cell adhesion molecule (hNCAM) revealed relatively small G418 and D37 grafts, with few hNCAM-ir fibers innervating the host striatum. In contrast, large hNCAM-ir grafts, and their processes, were visible in animals engrafted with either D17 or D24 cells. While all grafts contained TH-ir neurons, only D17 grafts were cytoarchitecturally arranged in a manner similar to what is characteristically seen with fVM implants, with dopaminergic cell bodies localized at the periphery of the grafts^36^.

After verifying that our modified differentiation protocol produced cells that are viable in the immunocompromised intact rat brain, we performed a long-term functional study. Rats with unilateral 6-OHDA-induced medial forebrain bundle (MFB) lesions confirmed by repeated d-amphetamine-induced rotations were transplanted with vehicle control or D17, D24, D37, or G418 cells (150,000 cells/μL; 3 μL; *n* = 9–11/group) and sacrificed 6 months post-injection. A summary table (Table 1) describes the histological and behavioral findings for each cell type and dosing group.

**Table 1:** Summary Table.

	Treatment							
Endpoint	G418	D37	D24	D17	D17,low dose	D17,medium dose	D17,high dose	D17,MFD
Behavioral recovery	Yes, by 6 MPI	No	Yes, by 6 MPI	Yes, by 4 MPI	No	Yes, by 6 MPI	Yes, by 4 MPI	Yes, by 4 MPI
TH ODU	0.33	0.13	0.30	0.46	0.09	0.13	0.36	0.51
TH-ir cells (%)	20,355(23.5%)	9,318(16.1%)	67,830(25.5%)	79,061(24.0%)	1,087(7.5%)	6,400(15.0%)	19,973(10.0%)	59,929(10.2%)
hKi-67-ir cells (%)	352 (0.6%)	0 (0.0%)	1,858 (0.6%)	3,412 (1.2%)	0 (0.0%)	532 (1.2%)	1,038 (0.4%)	2,402 (0.4%)
5-HT-ir cells (%)	n.d.	n.d.	n.d.	n.d.	n.d.	n.d.	n.d.	277 (0.04%)

Normalized TH ODU values range from a minimum of 0, representing the denervated striatum, to 1, representing the intact striatum.

*(%)* median percentage of estimated hNuclei-ir cells, *MPI* months post-injection, *n.d.* not determined.

**Fig. 3:**
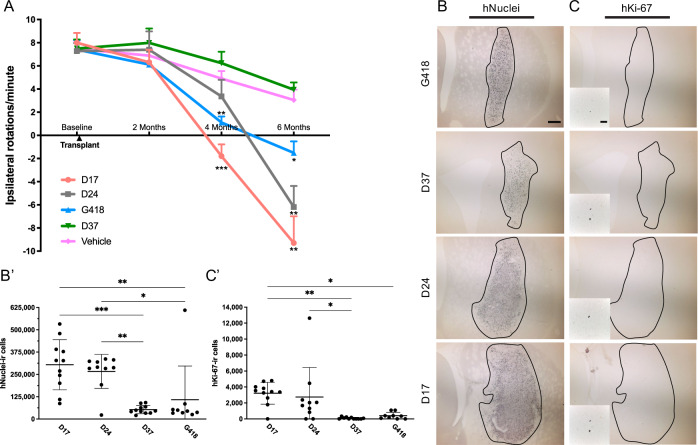
Graft survival and function. Time-based analysis of **A** d-amphetamine-induced rotations measured pre-operatively and at 2, 4, and 6 months post-engraftment. At 4 months post-transplantation, *P* < 0.0005 for D17 and *P* < 0.005 for G418; at 6 months post-transplantation, *P* < 0.0005 for D17 and D24 and *P* < 0.05 for G418. Data were analyzed by mixed ANOVA with Tukey’s adjustment; error bars are SEM. Comparisons were made to vehicle group. Representative graft sections stained for **B** hNuclei and **C** hKi-67 with graft borders indicated by black outline. Quantification by unbiased stereology of **B’** hNuclei-ir (*P* < 0.0001 D17 vs. D37/G418; *P* < 0.0005 and *P* < 0.005 for D24 vs. D37/G418, respectively) and **C’** hKi-67-ir cells (*P* < 0.05 for D17 vs. D37; *P* < 0.01 for D17 vs. G418; *P* < 0.05 for D24 vs. D37). Scale bar = 500 μM in **B**; 50 μM in **C** (inset). hNuclei estimates were analyzed by one-way ANOVA with Tukey’s adjustment; error bars represent SD. hKi-67 estimates were analyzed by Kruskal–Wallis test and Dwass–Steele–Critchlow-Fligner post hoc. *p < 0.05 **p < 0.001 ***p < 0.0001.

To demonstrate the functional capacity of each cell type, we performed d-amphetamine-induced rotation testing at baseline (10–11 weeks after 6-OHDA lesioning) and 2, 4, and 6 months post-transplantation (Fig. 3A). Hemiparkinsonian rats that received vehicle or D37 grafts failed to demonstrate functional recovery. A mixed-effects ANOVA with Tukey’s post hoc testing revealed that rats receiving D17, D24, or G418 cells exhibited significant (*P* < 0.005, *P* < 0.005, *P* < 0.05) recovery of motor asymmetry by 6 months post-injection. Additionally, animals receiving D17 grafts displayed full (*P* < 0.0005) normalization of rotations by 4 months post-injection.

To quantify survival of transplants of each cell type, we counted human-specific nuclei (hNuclei) in graft sections using unbiased stereology (Fig. 3B, B’). We estimated an average (±SD) of 304,303 ± 140,487 hNuclei-ir cells in the D17 group; 266,956 ± 95,419 in the D24 group; 52,623 ± 22,955 in the D37 group; and 108,093 ± 188,944 in the G418 group, representing 67.6, 59.3, 11.7, and 24.0%, respectively, of transplanted cells. A one-way ANOVA with Tukey’s post hoc adjustment demonstrated better engraftment and survival of grafts comprised of D17 (*P* < 0.005, *P* < 0.01) and D24 (*P* < 0.005, *P* < 0.05) cells compared to D37 and G418, respectively.

Excessive levels of proliferation would preclude any cell type from clinical use due to the increased risk for developing intracerebral tumors, teratomas or overgrowth of lineage restricted cells (e.g., neural progenitors). Stereological estimates of human-specific Ki-67 (hKi-67) revealed a median (±IQR) of 3412 ± 1391 hKi-67-ir cells in D17 grafts; 1858 ± 2275 in D24 grafts; 0 ± 180 in D37 grafts; and 352 ± 697 in G418 grafts, representing only 1.2, 0.6, 0.0, and 0.6% of hNuclei-ir cells, respectively (Fig. 3C, C’). We detected significant differences between total number of hKi-67-ir cells for D17 and G418 (*P* < 0.01) or D37 (*P* < 0.05) as well as between D24 and D37 (*P* < 0.05) and between D17 and D37 (*P* < 0.005) for the number as a proportion of hNuclei-ir cells using a Kruskal–Wallis rank-sum test with Dwass–Steele–Critchlow-Fligner post hoc test. These findings demonstrate that grafts of cells transplanted earlier in differentiation contained more proliferating cells post-implantation. Further, although we did not quantify graft volume, D17 and D24 grafts were qualitatively similar in size at 3 months and 6 months (Supplementary Fig. 3), suggesting that any volumetric expansion related to proliferation subsided soon after transplant surgery. Critically, there was no evidence of teratomas or outgrowths compressing neighboring brain regions.

**Fig. 4:**
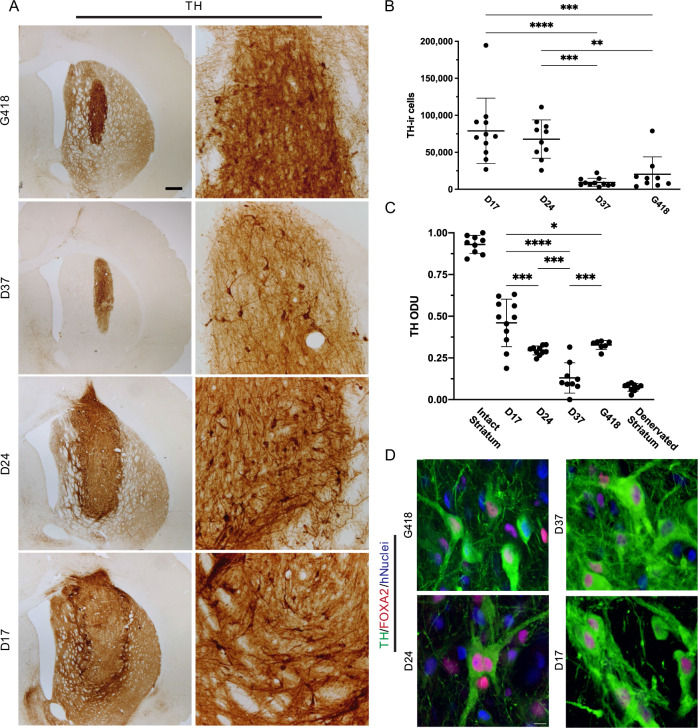
Visualization of dopaminergic phenotype in vivo. Representative graft-containing sections stained for **A** DAB-processed TH. Quantification of **B** TH-ir cells contained within grafts after 6 months in vivo (*P* < 0.0001 and *P* < 0.005 for D17 vs. D37/G418; *P* < 0.0005 and *P* < 0.01 for D24 vs. D37/G418, respectively). **C** Optical density of graft-derived TH-ir fibers. Significant *P* values were calculated for D17 vs. D24, D37, and G418 (*P* < 0.0005, *P* < 0.0001, *P* < 0.05); D24 vs. D37 (*P* < 0.001); and G418 vs. D37 (*P* < 0.0005). **D** Immunofluorescently triple-labeled for TH/FOXA2/hNuclei (green/red/blue). Scale bar A = 500 μM; D = 20 μM. *p < 0.05 **p < 0.001 ***p < 0.0001.

Next, we used stereology to estimate the number of TH-ir cells in each graft and found an average (±SD) of 79,061 ± 44,167 TH-ir cells in D17 grafts; 67,830 ± 25,944 in D24 grafts; 9318 ± 5523 in D37 grafts, and 20,355 ± 23,452 in G418 grafts, representing 24.0, 25.5, 16.1, and 23.5% of estimated hNuclei-ir cells, respectively (Fig. 4A, B). The TH-ir population was significantly larger in D17 (*P* < 0.0001, *P* < 0.005) and D24 (*P* < 0.0005 and *P* < 0.01) transplants compared to D37 and G418 transplants, respectively, by one-way ANOVA with Tukey’s post hoc test. There was also a significant difference between D17 and D37 (*P* < 0.05) for TH-ir cell yield.

To evaluate the ability of each cell type to reinnervate the host striatum with TH-ir axons, we measured TH optical density in the striatum, excluding the body of the graft. Using the TH-denervated striatum of vehicle-treated animals and the contralateral intact striatum as reference points, the data were rescaled from 0 to 1 based on the minimum and maximum values obtained, respectively, and converted to optical density units (ODU) (Fig. 4C). We calculated a mean (±SD) of 0.46 ± 0.14 ODU in D17-treated animals; 0.29 ± 0.03 ODU in D24-treated rats; 0.13 ± 0.09 ODU in D37-treated rats; and 0.33 ± 0.03 ODU in G418-treated rats. The D17 grafts had significantly more TH-ir processes than any other cell type (*P* < 0.0005, *P* < 0.0001, *P* < 0.05 compared to D24, D37, and G418, respectively), while both D24 (*P* < 0.001) and G418 (*P* < 0.0005) cells had significantly more than D37 transplants in a one-way ANOVA with Tukey’s post hoc adjustment. Together, these data demonstrate that cells transplanted earlier in development (namely D17) comprise populations enriched for TH and neurite outgrowth.

FOXA2 plays a critical role in the induction and maintenance of authentic mDA neurons^37,38^. We utilized immunofluorescent co-labeling to determine FOXA2 expression in hNuclei/TH-ir neurons (Fig. 4D) and found that most transplanted cells expressed FOXA2. A substantial subset of hNuclei/FOXA2-ir cells also expressed TH, confirming an authentic mDA phenotype.

### Long-range site-specific innervation

**Fig. 5:**
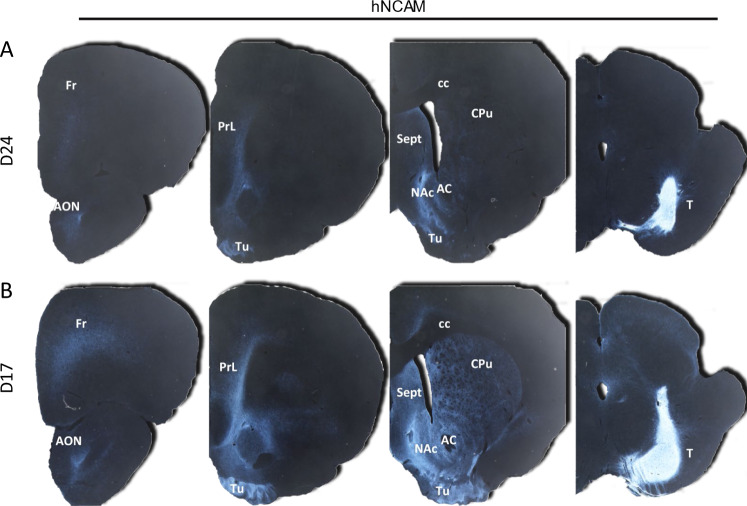
Long-range innervation of grafted cells transplanted in substantia nigra. Representative computer-inverted micrographs of hNCAM immunoreactivity in coronal sections spanning from the forebrain to the site of transplant in the substantia nigra. DAB-processed images were inverted and adjusted to show extent of innervation; all enhancements were applied to each sample in an identical fashion. AC anterior commissure, AON anterior olfactory nucleus, cc corpus callosum, CPu caudate/putamen, Fr frontal cortex, NAc nucleus accumbens, PrL prelimbic area, Sept septum, T transplant, Tu olfactory tubercle.

The ability to innervate over long distances will likely be necessary for successful translation of stem cell grafting to the human PD brain. To assess these capabilities in our cells, we grafted the most efficacious cell types, D17 or D24, into the SN and examined the long-range projections to their natural targets in the forebrain. At 6 months post-grafting, hNCAM immunoreactivity was evaluated in coronal sections to identify fibers emanating from the grafts and their targets (Fig. 5). Projections from D24 grafts primarily innervated A10 structures in the prelimbic cortex, olfactory tubercle, anterior olfactory nucleus, septum, and nucleus accumbens, with sparse fibers in the striatum, an A9 target. We observed markedly denser innervation of these same A9 and A10 targets in addition to the frontal cortex (A10) by D17 grafts. In both D17- and D24-grafted animals, we observed hNCAM-ir fibers in the most rostral brain regions examined (approximately 7-8 mm from the most rostral aspect of the graft in the SN), demonstrating the ability to project fibers over long-distances and, for D17 grafts, the ability to innervate areas normally innervated by A9 dopamine neurons.

### Dose response

The D17 grafts demonstrated the most robust efficacy, viability, and dopaminergic phenotypic expression without problematic proliferation, and were therefore chosen for further study. To determine an optimal dosing strategy, we titrated down the concentration of D17 cells from that used in the initial examination. Hemiparkinsonian athymic rats received 3 μL striatal transplants of the maximum feasible dose (MFD) of 150,000 cells/μL, High dose (40,000 cells/μL), Medium dose (10,000 cells/μL), Low dose (2500 cells/μL), or vehicle control (*n* = 8–11/group). Motor asymmetry was assessed every 2 months post-transplantation by d-amphetamine-induced rotations for 6 months, at which point rats were sacrificed and brains were assessed histologically.

**Fig. 6:**
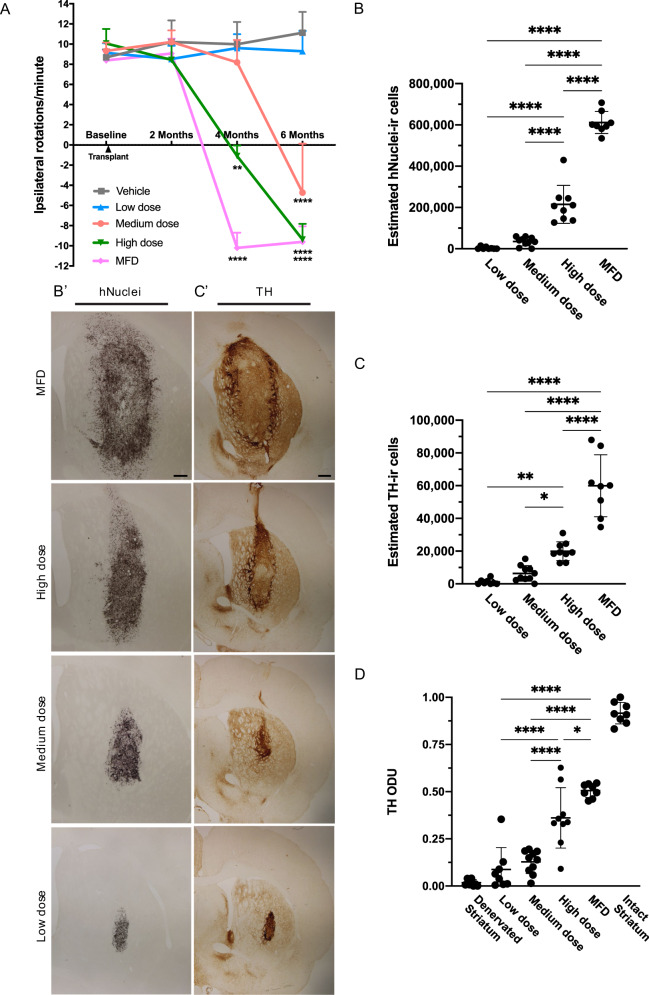
Quantitative analysis of function, survival, and innervation of D17 progenitors in vivo. Time-based analysis of **A** d-amphetamine-induced rotations measured pre-operatively and at 2, 4, and 6 months post-engraftment. At 4 months post-transplantation, *P* < 0.0001 for MFD and *P* < 0.0005 for high dose; at 6 months post-transplantation, *P* < 0.0001 for MFD and high dose; *P* < 0.005 for medium dose; analyzed by mixed ANOVA with Tukey’s adjustment. Comparisons were made to vehicle group. **B** Stereological estimates of hNuclei-ir cells (visualized in **B’**) contained in grafts of low, medium, high, or “maximum feasible” dose. *P* < 0.0001 for all comparisons by one-way ANOVA with Tukey’s adjustment. Stereological estimates of **C** TH-ir cells (visualized in **C’**) contained in grafts of low, medium, high, or “maximum feasible” dose. *P* < 0.0001 for MFD vs. all groups; *P* < 0.005 and *P* < 0.05 for high-dose vs. medium- and low-dose groups, respectively; analyzed by one-way ANOVA with Tukey’s adjustment. Quantification of **D** graft-derived TH optical density. One-way ANOVA with Tukey’s adjustment showed *P* < 0.0001 for MFD vs. medium and low doses and high vs. medium and low doses; *P* < 0.05 for MFD vs. high dose. One-way or mixed effects ANOVA with Tukey’s adjustment for histological or behavioral data, respectively; error bars represent SD or SEM for histological or behavioral data, respectively. Images for low-dose group are from a rat with substantial surviving graft. Scale bar = 500 μM. *p < 0.05 **p < 0.001 ***p < 0.0001.

We observed a clear dose response in all behavioral and histological analyses. Rats that received vehicle or low dose of transplanted cells failed to demonstrate functional recovery in the d-amphetamine-induced rotation test. However, a mixed-effects ANOVA with Tukey’s post hoc adjustment revealed that rats that received the medium (*P* = 0.002), high (*P* < 0.0001), or “maximum feasible” (*P* < 0.0001) dose displayed full normalization of motor asymmetry by 6 months after transplantation (Fig. 6A). Notably, grafts of the high (*P* = 0.0002) or “maximum feasible” (*P* < 0.0001) dose were effective in normalizing rotations as early as 4 months post-injection. Further, the extensive innervation in rats from the two highest dose groups resulted in over-compensation of d-amphetamine-induced rotation resulting in circling in the direction opposite to what was seen pre-grafting (Fig. 6A).

When we quantified hNuclei staining in the grafts (Fig. 6B, B’), the number of surviving cells directly correlated with dosage, as expected, with an estimated mean (±SD) 611,588 ± 53,377 surviving cells in MFD-treated animals; 214,898 ± 91,906 in high dose animals; 36,848 ± 18,816 in medium dose animals; and 4604 ± 5904 in low dose animals. Significant differences were calculated by a one-way ANOVA with Tukey’s post hoc test for MFD compared to low, medium, and high doses as well as high compared to medium and low doses (*P* < 0.0001 for all comparisons).

We also quantified the number of TH-ir cells within each graft (Fig. 6C, C’) using unbiased stereology. As expected, the number of TH-ir cells directly correlated with dosage, with an estimated mean (±SD) 59,929 ± 18,927 TH-ir cells in MFD grafts; 19,973 ± 5759 in high dose grafts; 6400 ± 4709 in medium dose grafts; and 1087 ± 1471 TH-ir cells in low-dose grafts, representing 10.2, 10.0, 15.0, and 7.5% of estimated hNuclei-ir cells, respectively. Significant differences were calculated for MFD (*P* < 0.0001) compared to low, medium, and high doses as well as high compared to medium (*P* = 0.03) and low (*P* = 0.002) doses using a one-way ANOVA with Tukey’s post hoc adjustment.

In order to evaluate the ability for each cell type to replenish the host tissue with TH-ir processes, we measured and processed TH optical density in the striatum in the same fashion as described above. The density of projections reinnervating the striatum correlated with dosage, with a mean (±SD) of 0.51 ± 0.04 ODU, 0.36 ± 0.16 ODU, 0.13 ± 0.06 ODU, and 0.09 ± 0.12 ODU calculated in the MFD, high, medium, and low dose groups, respectively (Fig. 6D). Significant differences were found when comparing MFD to low (*P* < 0.0001), medium (*P* < 0.0001), and high (*P* < 0.05) doses, as well as for high dose compared to medium (*P* < 0.0001) and low (*P* < 0.0001) doses with a one-way ANOVA and Tukey’s post hoc test.

**Fig. 7:**
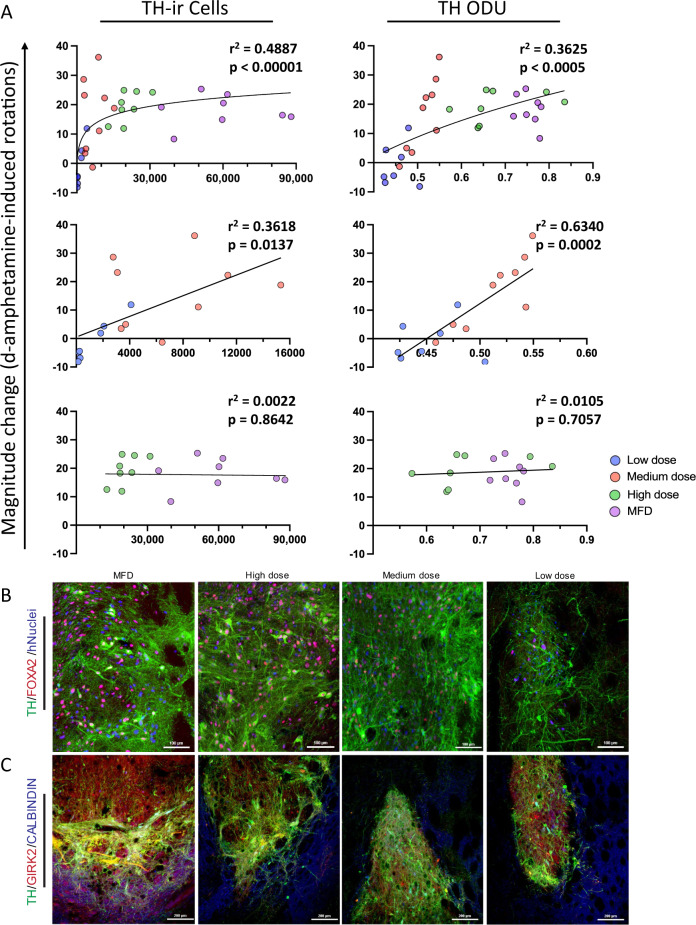
Correlations of dopaminergic phenotype with behavioral recovery and visualization of mDA subtype. **A** Estimated number of TH-ir cells and TH optical densitometric measurements plotted against the absolute value of the magnitude of change in net d-amphetamine-induced rotations and fitted with logarithmic regression curve. Linear regression for low/medium or high/“maximum feasible” doses and behavioral recovery. Representative images containing grafts of low, medium, high, and “maximum feasible” dose for immunofluorescent triple-labeled **B** hNuclei/TH/FOXA2 (blue/green/red) and **C** TH/GIRK2/Calbindin (green/red/blue).

Upon first assessment, the low dose group displayed no behavioral correction despite containing 4604 ± 5904 hNuclei-ir cells and 1087 ± 1471 TH-ir cells. However, further inspection revealed 5 rats with little-to-no surviving grafts that did not recover motor asymmetry. In contrast, rats with substantial surviving grafts (containing 1827; 2068; and 4100 TH-ir cells) recovered to varying degrees (18, 49, and 85% reduction in rotations, respectively) by 6 months post-transplantation. To further scrutinize the behavioral effect of different doses of D17 mDA progenitors, we plotted behavioral recovery against number of TH-ir cells and TH optical density (Fig. 7A). Given the non-linear quality of the data, we used logarithmic regression to assess these correlations. We found *r*^2^ = 0.3625 (*P* < 0.0005) for TH optical density and *r*^2^ = 0.4887 (*P* < 0.00001) for TH-ir cells indicating moderate correlations with functional recovery. When we partitioned the data into low/medium and high/MFD groups, we observed linear relationships in the low/medium groups for TH optical density (*r*^2^ = 0.6340; *P* < 0.0005) and TH-ir cells (*r*^2^ = 0.3618; *P* < 0.05). These analyses indicated that while there was a clear ceiling effect for both measures of dopaminergic phenotype, graft-derived innervation was a more robust indicator of overall graft function at lower doses.

### Characterization of mDA phenotype in vivo

To confirm mDA phenotype, we employed immunofluorescent triple-labeling of grafts at 6 months post-injection (Fig. 7B). We found that a majority of grafted cells expressed TH/FOXA2 with most TH-co-expressing cells localized to the borders of the graft. Additionally, in grafts of MFD-treated rats (*n* = 4), we found many cells expressing TH/GIRK2 (62.6 ± 2.9%), with a smaller population of TH/Calbindin-ir (31.8 ± 1.7%) cells (Fig. 7C), evincing both A9 and A10 dopaminergic subtypes, consistent with the long-range innervation patterns by D17 cells grafted to the SN. We also noted some GIRK2-ir cells that did not express TH (3.3 ± 1.2%), which may be of parabrachial or paranigral origin^39^.

### Proliferation, gliosis, and serotonergic contamination in the grafts

**Fig. 8:**
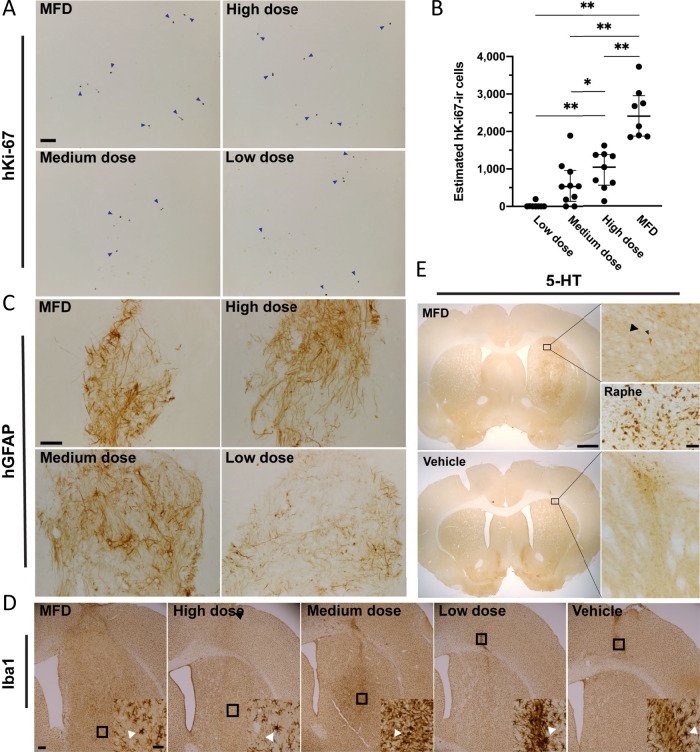
Non-dopaminergic cell types observed in grafts. Representative **A** micrographs of graft sections stained for hKi-67 and **B** stereological estimates for each group. **C** Representative images of graft sections stained for hGFAP (glia), **D**, Iba1 (microglia), and **E** 5-HT (serotonergic neurons). Scale bar **A** = 100 μM; **C** = 200 μM; **D** = 500 μM; **E** = 1 mm (**E**, inset) = 25 μM. *P* < 0.05 for medium vs. low dose; *P* < 0.005 for all other comparisons by Kruskal–Wallis test with Dwass, Steel, Critchlow-Fligner method. *p < 0.05 **p < 0.001.

Importantly, in agreement with our previous studies, we observed low levels of continued proliferation in the grafts after 6 months, as determined via unbiased stereology performed on sections stained for hKi-67 (Fig. 8A, B). We estimated 2402 ± 1006 hKi-67-ir cells in MFD grafts; 1038 ± 741 in high-dose grafts; 532 ± 745 in medium-dose grafts; and 0 ± 5 hKi-67-ir cells in low-dose grafts, representing 0.4, 0.4, 1.2, and 0.0% of estimated hNuclei-ir cells, respectively. We calculated significant differences for MFD compared to high (*P* = 0.003), medium (*P* = 0.004), and low (*P* = 0.003) dose as well as low compared to high (*P* = 0.003) and medium (*P* = 0.04) dose groups for total number of hKi-67-ir cells and for percentages of low compared to high and “maximum feasible” dose (*P* < 0.05) using Kruskal–Wallis and Dwass, Steel, Critchlow-Fligner method. Again, we report no evidence of teratoma formation.

To assess the degree of astrocytosis within the grafts, sections were stained with human-specific GFAP (Fig. 8C). We observed patterns of immunoreactivity, largely resembling long fibers coursing through the body of the graft with some astrocytic bodies, consistent with the *GLAST* expression detected by qPCR and similar to murine fVM grafts^40^. We evaluated Iba1-ir to determine whether there was an elevated microglial response to the xenotransplants. Generally Iba1-ir was not pronounced, except near the injection site in the cortex in close proximity to the craniotomy, site of dura puncture, and near the periphery of the graft, where there was slightly increased Iba1-ir and/or cells that had a morphology consistent with activated microglia. (Fig. 8D). It was noted in one animal in the medium dose group had slightly more intense Iba1-ir in the region that corresponded to the injection site. We found that D17 grafts contained very few serotonergic (5-HT) cells (Fig. 8E), with an estimated 277 ± 194 5-HT-ir cells (0.04% of estimated hNuclei-ir cells) in MFD grafts. These data collectively show an overall lack of outgrowth of off-target cell types or host gliosis.

## Discussion

In the present series of experiments, we established (1) a line of iPSCs and a differentiation process leading to the generation of mDA neurons suitable for clinical development; (2) that intrastriatal grafts of iPSC-derived mDA progenitors (cryopreserved on day 17 in vitro) in immunocompromised rats completely reverse 6-OHDA-induced motor asymmetry, survive in large numbers and densely reinnervate the host striatum, and are superior to grafts of cells cryopreserved on days 24 and 37; (3) that D17 progenitors mature and maintain the appropriate mDA lineage in vivo; (4) that D17 and D24 grafts placed in the substantia nigra exhibit long-range axonal growth to multiple host targets normally innervated by the mesotelencephalic dopamine system; (5) that higher doses of D17 progenitors provide faster and more complete functional recovery than lower doses with corresponding increases in cell survival and graft-derived TH innervation; and (6) that teratomas or excessive proliferation of cells are not observed when transplanting iPSC subjected to our differentiation protocol.

In our first experiment, grafts of mature (D37/G418) neurons clearly differed from transplants of immature neurons (D24) and progenitors (D17), both in terms of behavioral effects and regarding histological characteristics. The difference in graft size was apparent as early as 3 months post-injection based on hNCAM- and TH-immunostaining, with mature (D37/G418) neurons forming thin, pencil-shaped grafts, and younger (D17/D24) cells forming comparably large grafts. At 6 months post-grafting, we observed a robust dopaminergic phenotype in D17 and D24 grafts in comparison to D37 or G418, which was also reflected by a full reversal of d-amphetamine-induced motor asymmetry in D17- and D24-grafted rats. For all cell types and doses, grafted cells expressed TH/FOXA2, confirming that the maturation during the 6 months after grafting in vivo continued and resulted in mature mDA neurons derived from the implanted progenitors and immature neurons. When we grafted D17 and D24 cells intranigrally, we observed preferential innervation of both A9 and A10 targets over long distances. This is consistent with earlier observations with fVM and ESC-VM tissue^41,42^ and supported by our current findings of both TH/GIRK2-ir and TH/Calbindin-ir cells within the grafts. The ability of our grafted iPSC mDA cells to project fibers over long distances in the rat brain portends well for the translation of this therapy to the human putamen. Although GIRK2 and CALBINDIN are commonly used to differentiate A9 and A10 mDAs, it is important to note that these markers are arbitrary; and therefore in future studies sequencing and/or advanced multiplexing^43^ would be useful to more completely define these populations in our cell cultures. Notably, in our grafts, there are exceedingly rare mature cells that are inconsistent with optimal clinical benefit (i.e., those with characteristics of serotonergic, hindbrain, forebrain neurons, etc.) where intrastriatal transplants designed for DA replacement are desired for PD.

As expected, there were marked differences in outgrowth of graft-derived TH-immunoreactive fibers into the host striatum depending on the differentiation protocol used. Thus, rats grafted with D17, D24, and G418 cells exhibited TH-ir fibers covering the whole striatum, while rats grafted with D37 cells, which exhibited no graft-induced behavioral recovery, showed almost no graft derived TH-ir axons innervating the host. In fact, high magnification images revealed that though these grafts contained TH-ir cells and fibers, their axons ended abruptly upon reaching the outermost edges of the D37 grafts. With comparable numbers of TH-ir cells in D17 and D24 grafts as well as in D37 and G418 grafts, it is highly likely that the propensity for D17 and G418 cells to innervate the host underlies their function. Indeed, we saw similar behavioral outcomes in animals with large (67,800 TH-ir cells) D24 grafts as in animals with smaller (6,400 TH-ir cells) D17 grafts, presumably due to the similar reinnervation of the host striatum. Intriguingly, regression analyses showed not only a ceiling effect for number of D17 TH-ir cells and their processes but also that, at lower doses, innervation was more highly correlated than number of TH-ir cells with behavioral recovery. It is interesting to consider how this phenomenon will translate to humans, where the putamen (3.96 cm^3^ in PD patients^44^) is substantially larger than the rat striatum. On one hand, considerably more TH-ir cells and their processes will be necessary to produce clinical benefit; on the other, cells can be deposited at multiple sites along multiple needle tracts in an arrangement conducive to total reinnervation of the putamen, possibly without the diminishing returns associated with large grafts seen here in rats. In any case, the indication here that D17 mDA progenitors are effective across a wide range of doses suggests that clinicians will have some latitude in crafting a surgical approach and that careful studies will be required to optimize the dosing regimen in humans.

We rarely observed proliferating cells in grafts of mature cells (G418, D37), which is consistent with our previous observations^21,22^. While D17 and D24 grafts contained more hKi-67-ir cells than grafts of G418/D37 cells, the number of proliferating cells as a proportion of surviving grafted cells was low (<1000 per 100,000 hNuclei-ir in D17/D24 grafts), demonstrating that a purification step is not necessary to prevent undesirable cell proliferation. Further, hKi-67-ir cells were not present in clusters indicative of active cell division in any of our grafted rats. Another safety concern is the development of GIDs which have been reported in a subset of patients transplanted with fVM^9,14,45^, and it has been suggested that aberrant grafting of serotonergic neurons contributes to the development of GIDs. As further evidence of the safety of our cells, in our iPSC-derived grafts we did not find serotonergic neurons in numbers near those postulated to induce GIDs^46^.

Non-clinical transplantation studies using stem cell-derived mDA cells have focused on implanting progenitor and immature neuron developmental stages, which is consistent with the developmental age of fetal tissues used successfully in clinical trials^47^. It is difficult to directly compare the developmental stage of the cells used in different studies due to differences in the differentiation protocols, but it is interesting to note that those being adapted for translational use incorporate exposure to neuronal maturation factors such as BDNF, GDNF, TGF-β3, and/or DAPT^31,48–50^. Additionally, in studies where different developmental stages were directly compared, the conclusion has been that NURR1+ immature neurons were more efficacious than less mature progenitors^51,52^. By contrast, we have found with our protocol that D17 cells exposed to mDA patterning factors (SMAD inhibition, SHH, WNT, FGF8) and cryopreserved prior to NURR1 expression result in grafts that outperform the same cells cultured an additional week with maturation factors (D24, NURR1+/−). Cells at both maturational stages engraft and mature into mDA neurons in similar numbers, suggesting the performance disparity is not simply due to differences in proliferative potential (data not shown); rather it is more likely due to differences in innervation, A9 patterning, or other early mDA maturation signals received in vivo. Importantly, it is clear that D17 cells have been adequately patterned and do not require exposure to maturation factors before transplantation to “lock in” and exhibit DA neuron characteristics. This was evidenced by the percentage of TH+ cells in the D17 grafts being as high or higher than the percentage in D24 or D37 grafts (Table 1). While we were able to confirm the overall absence of aberrant cell types in the grafts (such as serotonergic neurons or proliferating cells), a significant portion of the cells in the grafts remains undefined. Further studies employing single-cell sequencing of grafted cells would be an ideal method to better understand the remaining non-dopaminergic neurons that comprise portions of these grafts.

The data presented here clearly support several prior reports that if the cells destined to become mDA neurons are too mature at the time of grafting to the striatum, they typically survive less well and have less marked behavioral effects. Our studies also demonstrate that relatively small grafts of D17 progenitors can give rise to dopaminergic innervation sufficient to elicit behavioral recovery in hemiparkinsonian rats. This suggests that a relatively small total number of cells might be required to be injected at a small number of locations in the striatum in each patient, which is favorable from a clinical safety standpoint. The early dose escalation studies in humans will inform future pivotal trials.

Other mDA progenitors that currently are being tested in clinical trials are derived from both ESCs (NCT04802733)^20^ and iPSCs (JMA-IIA00384, UMIN000033564)^48^. The preclinical data supporting these ongoing clinical transplantation trials in PD have shown that the transplanted cells have similar properties to those that we describe in the present study. We are optimistic about the therapeutic potential for each of these products and are interested to see how each cell source and manufacturing protocol performs when the cells are transplanted in PD patients. Now it will be important to design refined clinical neurosurgical protocols and immunosuppression regimens to achieve the full clinical benefit that is possible to attain with dopamine cell replacement therapy in carefully selected groups of PD patients.

## Methods

### Ethics statements

All animal procedures were performed with Institutional Animal Care and Use Committee approval from Rush University Medical Center; iPSCs in this study were generated from participants who provided written informed consent.

### Statistical analysis

Statistical analysis was performed in SAS (for stereological and behavioral outcomes in dosing study) or Prism (version 9.1.2, GraphPad). Graphs were made in Prism. Logarithmic and linear regression was performed in Prism. Data from immunohistochemical analyses were analyzed using a one‐way analysis of variance with Tukey’s test post hoc test, except for hKi-67 which was analyzed by Kruskal–Wallis test with Dwass, Steel, Critchlow-Fligner method post hoc. Behavioral data were analyzed by mixed effects analysis of variance with Tukey’s test post hoc test. Histological data were represented as mean ± SD except for hKi-67 (median ± IQR). Median percentages were reported for TH- or hKi-67-ir cells as proportion of hNuclei-ir cells. Rotations were reported as mean ± SEM.

### Cell differentiation

iPSCs were maintained in Essential 8 (E8) media (Thermo Fisher Scientific) on Vitronectin (Gibco) and cultured per FUJIFILM CELLULAR DYNAMICS INC. instructions (https://www.fujifilmcdi.com/product-literature/). Research use G418 neurons (iCell DopaNeurons, FUJIFILM Cellular Dynamics, Inc.) were derived as previously described^21,22^, utilizing an engineered iPSC line to allow G418 drug selection of neurons during the differentiation process, and with cryopreservation of the neurons on process day 38. For clinical development, a non-engineered iPSC line that had been reprogrammed using procedures and reagents appropriate for cell therapy development was selected and expanded into a master cell bank (MCB) and working cell bank (WCB) in a cGMP manufacturing facility (Waisman Biomanufacturing, Madison, WI). The iPSC-mDA differentiation protocol (Fig. 1A) was adjusted for this iPSC line, including simplification of SMAD signaling inhibition (200 nM LDN-193189, Reprocell) and shifting GSK-3 inhibition (1.65 μM CHIR99021, Reprocell) 1 day later, to process day 2, at a higher concentration adjusted for this timing. Raw materials were upgraded to be appropriate for clinical development, including the use of GMP grade Shh C25II (Biotechne, 100 ng/mL), BDNF (Biotechne, 20 μg/mL), GDNF (Biotechne, 20 μg/mL), and TGFβ3 (Biotechne, 10 μg/mL), in addition to the use of Purmorphamine (Cayman Chemical, 2 μM). D37 neurons were purified in-process using mitomycin C (Tocris; 150 ng/mL on process days 27 and 29) as previously described^22^, and were cryopreserved with CryoStor (Biolife Solutions) on process day 37. D17 progenitors were manufactured using the same differentiation process, except that progenitor aggregates were dissociated with CTS TrypLE Select Enzyme (Thermo) and cryopreserved on process day 17, without being exposed to maturation medium^29^ or mitomycin C treatment. D24 immature neurons were cryopreserved later in the process (process day 24), after being plated in maturation medium for one week, but without mitomycin C treatment. The cells used to compare iPSC DA maturation stages were produced in a research lab using the manufacturing process adapted for clinical translation. The D17 cells used for the dose-ranging study were made in a controlled, non-classified clean lab using the same process.

### qPCR

Cells were thawed and lysed with Buffer RLT Plus (Qiagen) containing 1:100 beta-Mercaptoethanol. Total RNA was extracted using a RNeasy Plus kit (Qiagen). cDNA was generated using a High Capacity RNA-to-cDNA Kit (ThermoFisher) with a 500 ng RNA input. Quantitative polymerase chain reaction (qPCR) was performed on a LightCycler480 (Roche) using TaqMan Gene Expression Master Mix (ThermoFisher), TaqMan assays (see Supplementary Table 1 for list of assays), and 2.5 ng cDNA input. Values are expressed as relative to glyceraldehyde-3-phosphate dehydrogenase (GAPDH). Three biological replicates were analyzed in technical triplicates for each time point.

### Fluidigm

Fluidigm was performed per manufacturer’s instructions. In brief, frozen cells were thawed as and single cells were sorted into 96 wp containing lysis buffer using a SONY SH800 sorter. After reverse transcription was performed (using 10× Superscript VILO Enzyme Mix, per manufacturer’s protocol (ThermoFisher), specific target amplification was performed using the primer pool provided by Fluidigm (employing Fluidigm custom primers) and the TaqMan PreAmp Master Mix (ThermoFisher). After the IFC plate was primed, SsoFast EvaGreen SuperMix (BioRad) and the diluted specific amplification product were loaded onto the IFC plate and run on the BioMark HD. Data was processed using the R library “fluidigmSC” and individual cell gene expression was graphed using GraphPad Prism.

### Flow cytometry

Cells were thawed as previously described^21^. Cells were centrifuged and stained with GhostDye510 (Tonbo Biosciences), fixed with 4% formaldehyde, and washed with wash buffer (2% FBS in DPBS). Cells were stained with primary antibodies in 1× BD Perm/Wash (BD Biosciences) +0.2% Triton X-100 (except for Map2 stain, which did not contain Triton X-100) at 4 °C (see Supplementary Table 2 for list of antibodies and dilutions), and labeled with secondary antibodies (where applicable) at room temp. Flow cytometry was performed on a MACSQuant® Analyzer 10 flow cytometer (Miltenyi Biotec). Three biological replicates were analyzed for each maturation time point. Gating strategies are depicted in Supplementary Fig. 4.

### Immunocytochemistry

Cells were thawed, seeded at 170,000 cells/well to 96-well plates, cultured overnight, and fixed with 4% formaldehyde. Cells were stained with primary antibodies in stain buffer (2% FBS, 2% Donkey Serum, 0.2% Triton X-100 in DPBS) at 4 °C (see Supplementary Table 2 for list of antibodies and dilutions), and labeled with secondary antibodies (where applicable) and Hoechst (ThermoFisher) at room temp. Cells were analyzed on an ImageXpress High Content Imager (Molecular Devices) at ×10 magnification. Three biological replicates were analyzed for each time point.

### Lesion induction and transplant

Female athymic nude (rnu) rats were acclimated for one week following reception. At 9‐10 weeks of age, (170–200 g) rats received unilateral injections of 6‐OHDA (15 mg in 3 μL 0.5% ascorbic acid) to the right MFB (Anterior/Posterior [AP]: −4.0 mm; Medial/Lateral [ML]: −1.3 mm from bregma, Dorsal/Ventral [DV]: −7.0 mm from dura). Animals with confirmed lesions by 10 weeks post-lesion received striatal (AP: +0.5 mm; ML: ±3.0 mm from bregma, DV: −5.3 mm from dura) injections of iPSC‐mDA cells (*n* = 8–11/group) and were sacrificed at 3 or 6 months post‐transplantation. Cryopreserved cells were thawed and cells counted via trypan blue exclusion. The cells were centrifuged and resuspended in a GMP-grade balanced salt solution at the appropriate densities for injection. Intranigral grafts were placed at AP: +0.5 mm; ML: −3.0 mm from bregma, DV: −5.0 mm from dura. In all experiments, injection volume was 3 μL. A concentration of 150,000 cells/μL was used in the cellular maturity comparison and intranigral experiments, and 2500, 10,000, 30,000, or 150,000 cells/μL was used for the dose-ranging experiment.

### d-amphetamine-induced rotations

Animals received intraperitoneal injections of d-amphetamine (2.5 mg/kg, Sigma), placed in harnesses in semi-opaque chambers, and connected to a Rotometer system (San Diego Instruments). Net ipsilateral (clockwise) rotations for the time period 10–40 min following d-amphetamine administration were reported.

### Tissue processing

Tissue was processed and immunohistological and stereological analyses were performed as previously described^22^. Briefly, rats were anesthetized with a ketamine/xylazine mixture and perfused with normal saline followed by 4% paraformaldehyde. Brains were removed, placed in a sucrose gradient, and sectioned at 40 μM on a sliding microtome. Free-floating sections were stained using antibody concentrations for immunofluorescent triple labeling or DAB processing listed in Supplementary Table 2. Sections were mounted on glass gelatin-coated slides, coverslipped, and imaged.

### Stereology

Coverslipped slides were analyzed by unbiased stereology (StereoInvestigator v10.40, MBF biosciences). For cellular maturity comparison experiment, 5.22% of total graft area was probed for TH, hNuclei, or hKi-67 in half series (1/12 serial sections) of stained tissue. For dose-ranging experiment, 5.22% of TH-ir and hNuclei-ir grafts, 28.4% of hKi-67-ir grafts, or 20.3% of 5-HT-ir grafts were probed in half series (1/12 serial sections) of stained tissue. For animals in low or medium dose groups with Gundersen *m* = 1 coefficient of error ≥0.45 or where no cells were counted in either hNuclei- or TH-stained sections, an additional half series (1/12 serial sections) was stained and re-probed using the same parameters. Estimates were then calculated for the full series (1/6 serial sections) and the results were averaged.

### Optical density

Grayscale images of 7 (center of graft ±3) coronal sections stained for TH were analyzed for each animal. In each section, a contour was drawn around the striatum, excluding the body of the graft, and mean pixel intensity of the area was recorded using ImageJ. Values were averaged for each animal and the data were rescaled considering the minimum point of the denervated striatum as 0 and the maximum point of the intact striatum as 1. Data sets for cellular maturity comparison and dose ranging experiments were rescaled separately.

### mDA subtype quantification

Graft sections from *n* = 4 MFD animals were stained for TH/GIRK2/CALBINDIN and imaged by a Nikon Eclipse Ti2 confocal microscope with a Nikon A1RHD camera using the NIS Elements AR software (version 5.10.01) and stored as.tiff files. Markers in 53–80 cells in each graft were quantified from *z*-stacks using ImageJ (version 1.53a).

### Reporting summary

Further information on research design is available in the Nature Research Reporting Summary linked to this article.

## Supplementary information

Supplementary Tables and Figures

REPORTING SUMMARY

## Data Availability

Data are available from authors upon reasonable request.
