# Supplementary material for: Optimizing maturity and dose of iPSC-derived dopamine progenitor cell therapy for Parkinson’s disease
Source: NPJ Regen Med. 2022 Apr 21;7:24. doi: 10.1038/s41536-022-00221-y (PMC9023503; doi:10.1038/s41536-022-00221-y)
Supplement: Supplementary file 1 — Supplementary Tables and Figures [file 41536_2022_221_MOESM1_ESM.pdf]

Supplementary Figure 1 Single cell gene expression in vitro

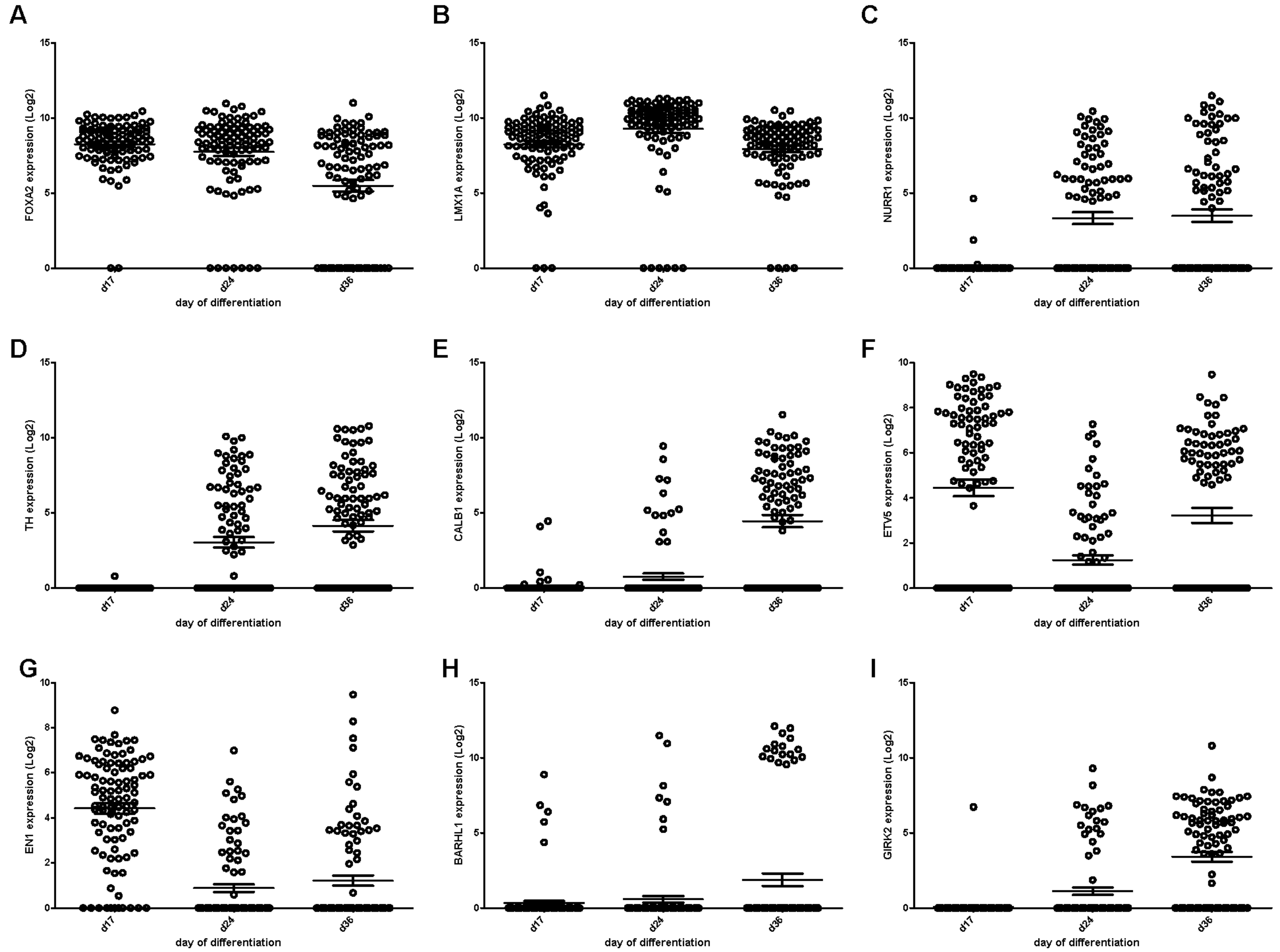

Single cell qPCR (Fluidigm) comparing mRNA expression at iPSC-mDA differentiation Days 17, 24, and 36 of target markers for A) FoxA2, B) LMX1A, C) NURR1, D) TH, E) CALB1, F) ETV5, G) EN1, H) BARHL1, and I) GIRK2. 96 individual cells were evaluated for each process timepoint. Log2 expression values for each cell represented as a single mark on the graph. Error bars are SEM.

Supplementary Figure 2 Visualization of protein expression *in vitro*

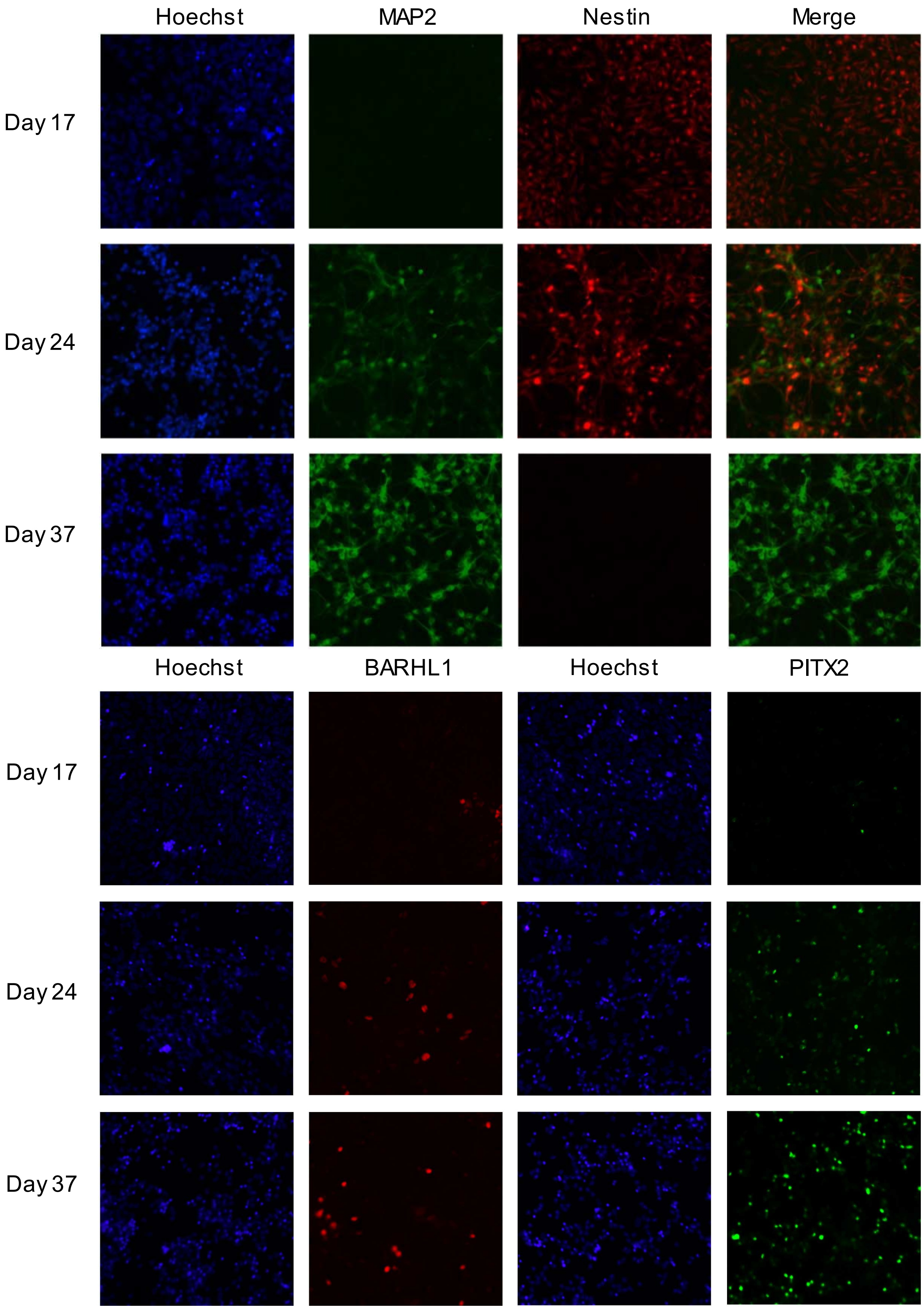

Immunocytochemistry comparing immunoreactive populations at iPSC-mDA differentiation Days 17, 24, and 37 of mDA target and off-target markers. Images are representative of three biological replicates analyzed for each timepoint.

Supplementary Figure 3 Short-term engraftment

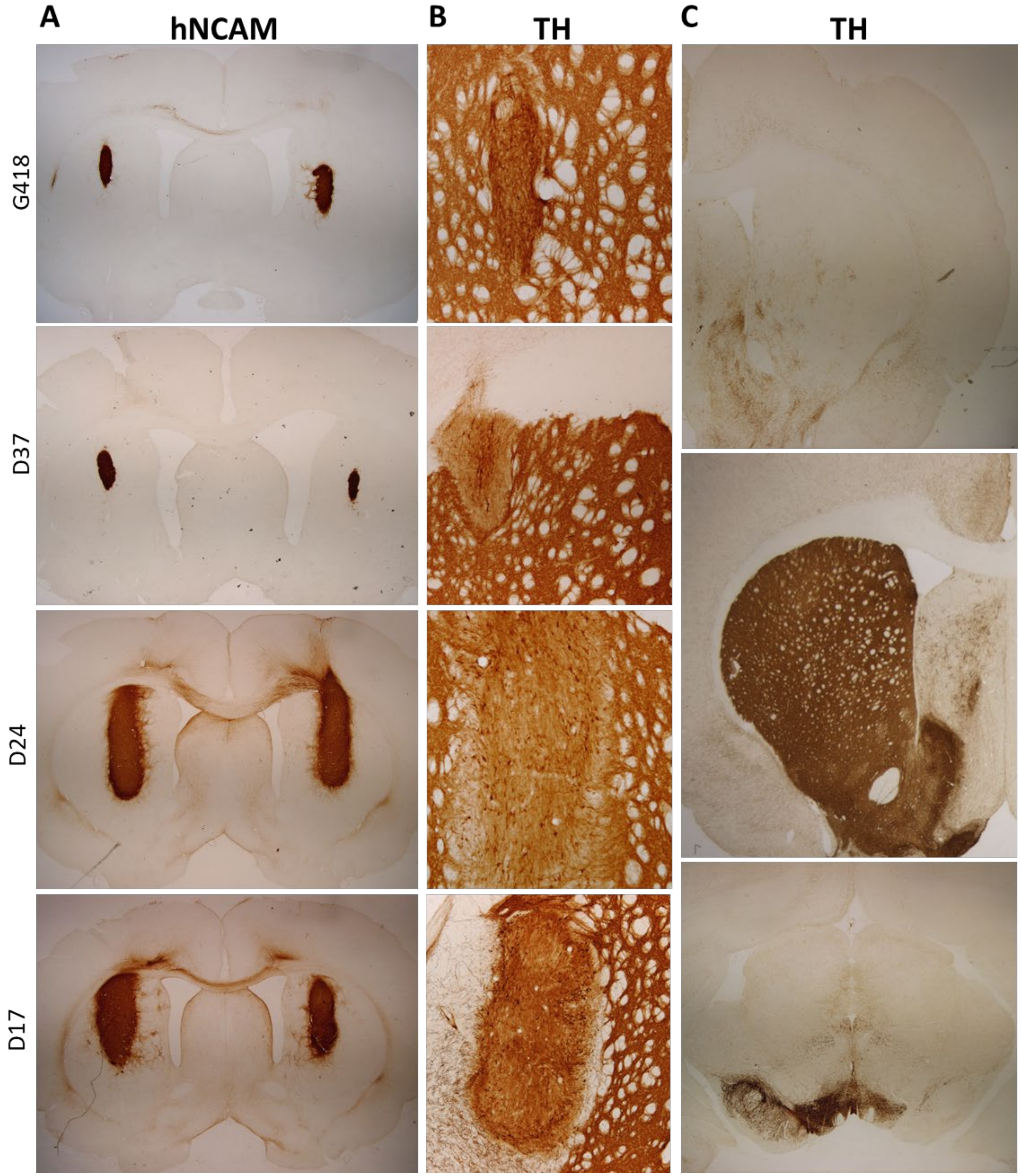

Coronal sections containing bilateral G418, D37, D24, or D17 striatal grafts in intact rats 3 months post-injection stained for (**A**) hNCAM or (**B**) TH. Representative TH-denervated (top) and intact (middle) striatum; substantia nigra of 6-OHDA-lesioned rat treated with vehicle control (bottom) (**C**).

Supplementary Figure 4 Gating strategies

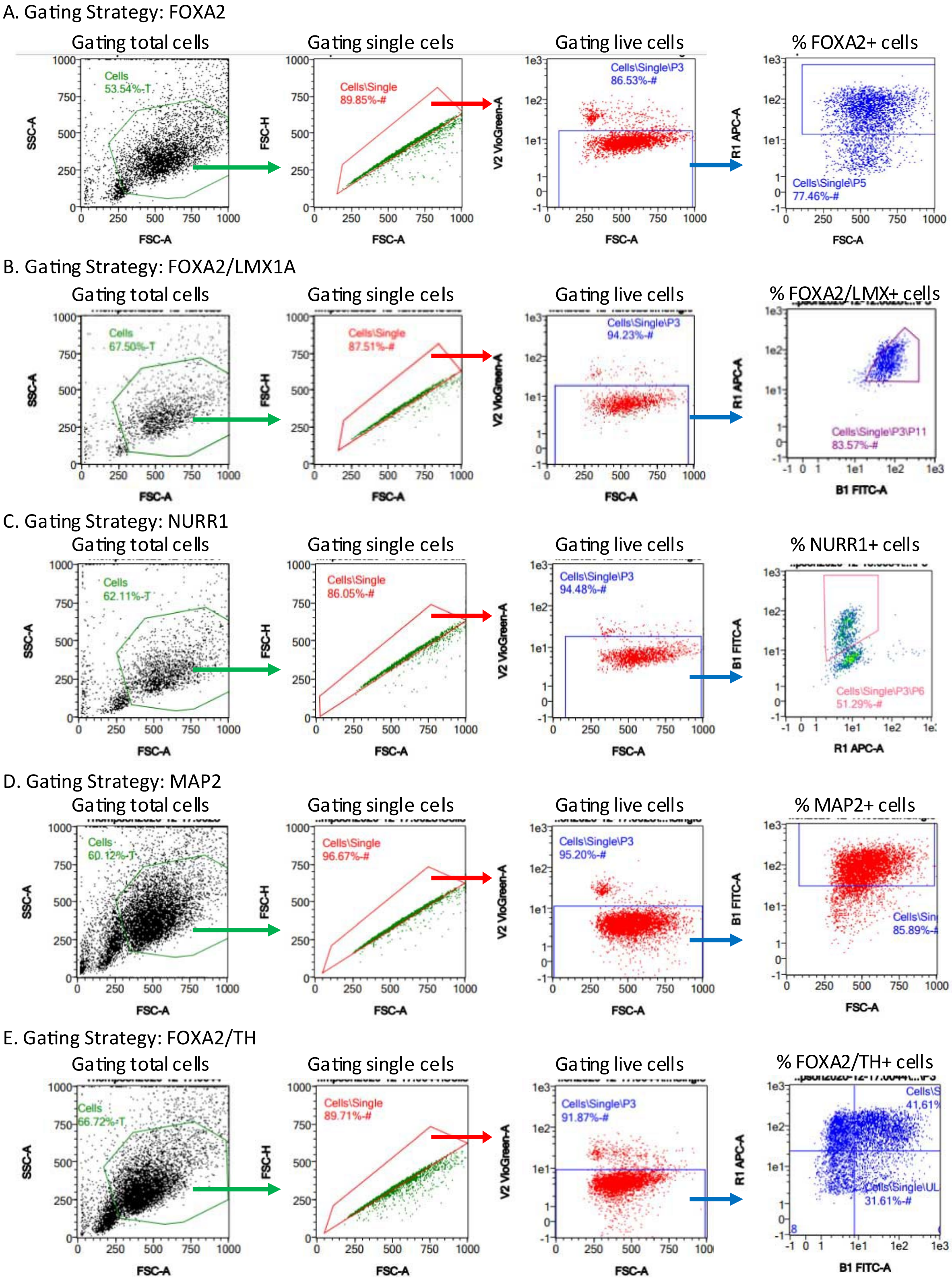

Gating strategies for FACS experiments in Figure 2A: FOXA2 (A), FOXA2/LMX1A (B), NURR1 (C), MAP2 (D), FOXA2/TH (E).

Supplementary Table 1 Table of qPCR primers

Gene	TaqMan Assay
OTX2	Hs00222238_m1
FOXA2	Hs00232764_m1
LMX1A	Hs00898455_m1
EN1	Hs00154977_m1
NURR1/NR4A2	Hs00428691_m1
PITX3	Hs01013935_m1
TH	Hs00165941_m1
SLC6A3 / DAT	Hs00997374_m1
GIRK2 / KCNJ6	Hs01040524_m1
CALB	Hs00191821_m1
ETV5	Hs00927557_m1
CNPY1	Hs01073160_m1
SPRY1	Hs01083036_s1
DBX1	Hs01380082_m1
PHOX2A	Hs00605931_m1
HB9 / MNX1	Hs00907365_m1
CHAT	Hs00758143_m1
VGLUT1	Hs00220404_m1
GAD1 / GAD67	Hs01065893_m1
SLC6A4 / SERT	Hs00169010_m1
GLAST / SLC1A3	Hs00188193_m1
S100B	Hs00902901_m1
CD44	Hs01075864_m1
PITX2	Hs04234069_mH
BARHL1	Hs01063929_m1
HOXA2	Hs00534579_m1
LHX2	Hs00180351_m1
FOXG1	Hs01850784_m1
PAX6	Hs00242217_m1
POU4F1 / BRN3A	Hs00366711_m1
SOX1	Hs01057642_s1
DCX	Hs01035496_m1
NeuN / RBFOX3	Hs01370653_m1
GAPDH	4333764F

Supplementary Table 2 Table of antibodies

Marker	Vendor, Catalog	Dilution	LifeTech Alexa Fluor® (1:1000)
Flow Cytometry			
Rabbit - FOXA2	Cell Signaling, 8186	1:500	A21244
Mouse - FOXA2	Abcam, ab60721	1:10,000	A31571
Rabbit - LMX1	Millipore, AB10533	1:10,000	A11008
Mouse - NURR1	ThermoFisher, MA1-195	1:1,000	A21202
Mouse - MAP2 + Alexa488	Millipore, MAB3418X	1:1,000	NA
Mouse - Nestin +Alexa647	BD, 560393	1:20	NA
Mouse - TH	Sigma, T2928	1:8500	A21121
Immunocytochemistry			
Mouse - FOXA2	Abcam, ab60721	1:10,000	A21131
Rabbit - LMX1	Millipore, AB10533	1:5,000	A21244
Mouse - NURR1	ThermoFisher, MA1-195	1:1,000	A21131
Mouse - TH	Sigma, T2928	1:10,000	A21202
Mouse - MAP2 + Alexa488	Milipore, MAB3418X	1:1,000	NA
Mouse - Nestin +Alexa647	BD, 560393	1:20	NA
Rabbit - BARHL1	Novus Biologicals, NBP1-86513	1:1,000	A31573
Sheep - PITX2	R&D Systems, AF7388	1:1,000	A11015
Immunohistochemistry			DAB or IFC
Mouse – hNuclei	Millipore, MAB1281	1:800	DAB
Mouse – hNuclei	Millipore, MAB1281	1:400	IFC
Rabbit – TH	Pelfreez, P40141	1:1,000	DAB
Rabbit – TH	Pelfreez, P40141	1:500	IFC
Mouse – hKi67	Cell Signaling, 9027S	1:1,000	DAB
Rabbit – 5-HT	Millipore, S5545	1:10,000	DAB
Rabbit – Iba1	Wako, 019-19741	1:1,000	DAB
Rabbit – GFAP	SC123, Y40420	1:2,000	DAB
Goat – FOXA2 (HNF-3β)	R&D Systems, AF2400	1:200	IFC
Mouse – FOXA2	Abcam, ab60721	1:200	IFC
Goat – GIRK2 (K_{ir}3.2)	Abcam, ab65096	1:300	IFC
Mouse – Calbindin	Sigma, C9848	1:300	IFC
Horse anti-Mouse	Vector Labs, BA-2001	1:200	NA
Goat anti-Rabbit	Vector Labs, BA-1000	1:200	NA
Donkey anti-Sheep AF-488	Invitrogen, A-11015	1:200	NA
Donkey anti-Rabbit AF-488	Invitrogen, A-21206	1:200	NA
Donkey anti-Rabbit AF-555	Invitrogen, A-31572	1:200	NA
Donkey anti-Mouse AF-647	Invitrogen, A-31571	1:200	NA
Donkey anti-Mouse AF-488	Invitrogen, A-21202	1:200	NA
Donkey anti-Goat-AF-488	Invitrogen, A-11055	1:200	NA
Donkey anti-Goat-AF-555	Invitrogen, A-21432	1:200	NA

Supplementary Table 3 Significance of qPCR (Figure 1)

	D17 vs. D24	D17 vs. D37	D24 vs. D37
otx2	*	***	***
foxa2	***	***	*
lmx1a	*	ns	*
en1	**	**	ns
pitx3	***	ns	***
nurr1	ns	ns	ns
TH	ns	ns	ns
Dat	ns	ns	ns
Girk2	ns	ns	ns
Calb	ns	ns	ns
etv5	***	***	ns
cnpy1	ns	ns	ns
spry1	ns	ns	ns
Dbx1	ns	ns	ns
pitx2	ns	ns	ns
barhl	ns	ns	ns
hoxa2	ns	ns	ns
Lhx2	ns	ns	*
Foxg1	*	*	ns
Pax6	ns	ns	ns
BRN3a	ns	ns	ns
Phox2a	*	**	ns
HB9	*	*	ns
Chat	ns	**	ns
Vglut1	ns	ns	ns
Gad1	*	ns	*
Sert	***	ns	***
Glast	*	**	ns
S100b	ns	ns	ns
CD44	ns	ns	ns
Sox1	ns	ns	ns
Dcx	ns	**	ns
Neun	ns	**	*
Nkx2.1	ns	ns	ns
Eomes	ns	ns	ns

One-way ANOVA with Bonferroni post-hoc test. (* $P < 0.05$, ** $P < 0.01$, *** $P < 0.001$).
