# Supplementary material for: Optimizing maturity and dose of iPSC-derived dopamine progenitor cell therapy for Parkinson’s disease
Source: NPJ Regen Med. 2022 Apr 21;7:24. doi: 10.1038/s41536-022-00221-y (PMC9023503; doi:10.1038/s41536-022-00221-y)
Supplement: Supplementary file 2 — REPORTING SUMMARY [file 41536_2022_221_MOESM2_ESM.pdf]

nature portfolio

Corresponding author(s): DBPR NPJREGENMED-00707R

Last updated by author(s): Nov 20, 2021

Reporting Summary

Nature Portfolio wishes to improve the reproducibility of the work that we publish. This form provides structure for consistency and transparency in reporting. For further information on Nature Portfolio policies, see our Editorial Policies and the Editorial Policy Checklist.

Statistics

For all statistical analyses, confirm that the following items are present in the figure legend, table legend, main text, or Methods section.

n/a	Confirmed	
☐	☒	The exact sample size (n) for each experimental group/condition, given as a discrete number and unit of measurement
☐	☒	A statement on whether measurements were taken from distinct samples or whether the same sample was measured repeatedly
☐	☒	The statistical test(s) used AND whether they are one- or two-sided<br>*Only common tests should be described solely by name; describe more complex techniques in the Methods section.*
☐	☒	A description of all covariates tested
☐	☒	A description of any assumptions or corrections, such as tests of normality and adjustment for multiple comparisons
☐	☒	A full description of the statistical parameters including central tendency (e.g. means) or other basic estimates (e.g. regression coefficient) AND variation (e.g. standard deviation) or associated estimates of uncertainty (e.g. confidence intervals)
☐	☒	For null hypothesis testing, the test statistic (e.g. F, t, r) with confidence intervals, effect sizes, degrees of freedom and P value noted<br>*Give P values as exact values whenever suitable.*
☒	☐	For Bayesian analysis, information on the choice of priors and Markov chain Monte Carlo settings
☒	☐	For hierarchical and complex designs, identification of the appropriate level for tests and full reporting of outcomes
☒	☐	Estimates of effect sizes (e.g. Cohen's d, Pearson's r), indicating how they were calculated

Our web collection on statistics for biologists contains articles on many of the points above.

Software and code

Policy information about availability of computer code

Data collection	*Provide a description of all commercial, open source and custom code used to collect the data in this study, specifying the version used OR state that no software was used.*
Data analysis	Statistical analysis was performed in SAS or Prism (version 9.1.2, GraphPad)

For manuscripts utilizing custom algorithms or software that are central to the research but not yet described in published literature, software must be made available to editors and reviewers. We strongly encourage code deposition in a community repository (e.g. GitHub). See the Nature Portfolio guidelines for submitting code & software for further information.

Data

Policy information about availability of data

All manuscripts must include a data availability statement. This statement should provide the following information, where applicable:

- Accession codes, unique identifiers, or web links for publicly available datasets
- A description of any restrictions on data availability
- For clinical datasets or third party data, please ensure that the statement adheres to our policy

Data are available from authors upon reasonable request.

Field-specific reporting

Please select the one below that is the best fit for your research. If you are not sure, read the appropriate sections before making your selection.

☒ Life sciences ☐ Behavioural & social sciences ☐ Ecological, evolutionary & environmental sciences

For a reference copy of the document with all sections, see nature.com/documents/nr-reporting-summary-flat.pdf

Life sciences study design

All studies must disclose on these points even when the disclosure is negative.

Sample size	Samples sizes were determined based on historical data.
Data exclusions	*Describe any data exclusions. If no data were excluded from the analyses, state so OR if data were excluded, describe the exclusions and the rationale behind them, indicating whether exclusion criteria were pre-established.*
Replication	Three biological replicates were analyzed for qPCR, FACS, ICC
Randomization	Treatment assignment following lesion induction was based on lesion severity; briefly, animals were assigned to groups to achieve similar mean +/- standard deviation baseline d-amphetamine-induced rotations.
Blinding	All staff were blinded to group allocation during data collection and analysis for animal and in vitro experiments

Reporting for specific materials, systems and methods

We require information from authors about some types of materials, experimental systems and methods used in many studies. Here, indicate whether each material, system or method listed is relevant to your study. If you are not sure if a list item applies to your research, read the appropriate section before selecting a response.

Materials & experimental systems

n/a	Involved in the study	
☐	☒	Antibodies
☐	☒	Eukaryotic cell lines
☒	☐	Palaeontology and archaeology
☐	☒	Animals and other organisms
☒	☐	Human research participants
☒	☐	Clinical data
☒	☐	Dual use research of concern

Methods

n/a	Involved in the study	
☒	☐	ChIP-seq
☐	☒	Flow cytometry
☒	☐	MRI-based neuroimaging

Antibodies

Antibodies used

Marker Vendor, Catalog
Flow Cytometry
Rabbit - FOXA2 Cell Signaling, 8186
Mouse - FOXA2 Abcam, ab60721
Rabbit - LMX1 Millipore, AB10533
Mouse - NURR1 ThermoFisher, MA1-195
Mouse - MAP2 + Alexa488 Millipore, MAB3418X
Mouse - Nestin +Alexa647 BD, 560393
Mouse - TH Sigma, T2928
Immunocytochemistry
Mouse - FOXA2 Abcam, ab60721
Rabbit - LMX1 Millipore, AB10533
Mouse - NURR1 ThermoFisher, MA1-195
Mouse - TH Sigma, T2928
Mouse - MAP2 + Alexa488 Milipore, MAB3418X
Mouse - Nestin +Alexa647 BD, 560393
Rabbit - BARHL1 Novus Biologicals, NBP1-86513
Sheep - PITX2 R&D Systems, AF7388
Immunohistochemistry
Mouse – hNuclei Millipore, MAB1281
Mouse – hNuclei Millipore, MAB1281
Rabbit – TH Pelfreez, P40141
Rabbit – TH Pelfreez, P40141

Mouse – hKi67 Cell Signaling, 9027S
Rabbit – 5-HT Millipore, S5545
Rabbit – Iba1 Wako, 019-19741
Rabbit – GFAP SC123, Y40420
Goat – FOXA2 (HNF-3[illegible]) R&D Systems, AF2400
Mouse – FOXA2 Abcam, ab60721
Goat – GIRK2 (Kir3.2) Abcam, ab65096
Mouse – Calbindin Sigma, C9848
Horse anti-Mouse Vector Labs, BA-2001
Goat anti-Rabbit Vector Labs, BA-1000
Donkey anti-Sheep AF-488 Invitrogen, A-11015
Donkey anti-Rabbit AF-488 Invitrogen, A-21206
Donkey anti-Rabbit AF-555 Invitrogen, A-31572
Donkey anti-Mouse AF-647 Invitrogen, A-31571
Donkey anti-Mouse AF-488 Invitrogen, A-21202
Donkey anti-Goat-AF-488 Invitrogen, A-11055
Donkey anti-Goat-AF-555 Invitrogen, A-21432

Validation: Each antibody was validated according to manufacturers' protocols.

Eukaryotic cell lines

Policy information about cell lines

Cell line source(s)	FCDI GMP grade iPSC
Authentication	STR
Mycoplasma contamination	Cell lines tested negative for mycoplasma
Commonly misidentified lines (See ICLAC register)	N/A

Animals and other organisms

Policy information about studies involving animals; ARRIVE guidelines recommended for reporting animal research

Laboratory animals	Female nude rats, age 7-9 weeks at beginning of study
Wild animals	*Provide details on animals observed in or captured in the field; report species, sex and age where possible. Describe how animals were caught and transported and what happened to captive animals after the study (if killed, explain why and describe method; if released, say where and when) OR state that the study did not involve wild animals.*
Field-collected samples	*For laboratory work with field-collected samples, describe all relevant parameters such as housing, maintenance, temperature, photoperiod and end-of-experiment protocol OR state that the study did not involve samples collected from the field.*
Ethics oversight	Animal experiments were approved by the Rush University Medical Center IACUC (16-084, 18-023)

Note that full information on the approval of the study protocol must also be provided in the manuscript.

Flow Cytometry

Plots

Confirm that:

☐ The axis labels state the marker and fluorochrome used (e.g. CD4-FITC).

☐ The axis scales are clearly visible. Include numbers along axes only for bottom left plot of group (a 'group' is an analysis of identical markers).

☐ All plots are contour plots with outliers or pseudocolor plots.

☐ A numerical value for number of cells or percentage (with statistics) is provided.

Methodology

Sample preparation	Cells were thawed and centrifuged and stained with GhostDye510 (Tonbo Biosciences), fixed with 4% formaldehyde, and washed with wash buffer before staining with primary antibodies
Instrument	MACSQuant® Analyzer 10 flow cytometer (Miltenyi Biotec)
Software	MACSQuantify

Cell population abundance

No sorting was performed

Gating strategy

FSC/SSC was performed to gate the single cells, then single cells were sorted on live/dead stain, then stained populations were analyzed

☐ Tick this box to confirm that a figure exemplifying the gating strategy is provided in the Supplementary Information.
